# Co-occurrence networks reveal candidate AMF–microbe assemblages for generalist and crop-specific inocula

**DOI:** 10.1007/s00572-026-01257-4

**Published:** 2026-04-13

**Authors:** Mathieu Delaeter, Papa Mamadou Sitor Ndour, Benoit Tisserant, Béatrice Randoux, Lobna Abdellatif, Franck Stefani, Maryline Magnin-Robert, Anissa Lounès-Hadj Sahraoui

**Affiliations:** 1 Unité de Chimie Environnementale et Interactions sur le Vivant (UCEIV, UR 4492), Université du Littoral Côte d’Opale, 50 rue Ferdinand Buisson, Calais CEDEX, 62228 France; 2 Ottawa Research and Development Centre, Agriculture and Agri-Food Canada, Ottawa, ON Canada

**Keywords:** AMF, Co-occurrence network, Inoculum, Wheat

## Abstract

Arbuscular mycorrhizal fungi (AMF) are widespread root symbionts increasingly used as bioinoculants. Because symbiotic efficiency depends on interactions with other plant-beneficial microbes, identifying compatible taxa and positive interaction patterns across hosts and niches could improve the design of crop-specific AMF-based inocula. Using amplicon sequencing and co-occurrence network analyses, microbial communities (AMF, fungi and bacteria) from the rhizosphere and roots of wheat were characterized and compared with those of two highly mycotrophic plant species: leek (monocotyledon) and clover (dicotyledon). Results showed that AMF diversity associated with wheat roots was 1.37- and 1.24-fold lower than that observed in leek and clover, respectively. Across all plant species, *Glomus* and *Rhizophagus* taxa dominated root-associated communities, whereas *Diversispora* prevailed in the rhizosphere. In contrast, wheat harboured the highest bacterial and fungal richness compared with leek and clover, whereas leek and clover roots were enriched in several functional bacterial groups, including nitrogen-fixing bacteria, denitrifying and nitrifying bacteria and plant growth promoting bacteria. Co-occurrence network analyses revealed niche partitioning with bacterial interactions predominant in roots and fungal interactions in the rhizosphere, but with fewer and less-positive connections in wheat than in leek and clover. AMF showed positive associations with beneficial bacterial taxa (e.g. *Rhizobium*, *Pseudomonas*, *Streptomyces*) and fungal taxa (e.g. *Serendipita*), with interaction patterns varying among plant species. Together, these results demonstrate that plant identity and niche jointly shape the diversity and interaction patterns of root and rhizosphere-associated microbial communities, and they highlight specific AMF-microbe assemblages as promising candidates for the development of generalist or crop-specific AMF-based inocula.

**Supplementary Information:**

The online version contains supplementary material available at 10.1007/s00572-026-01257-4.

## Introduction

Arbuscular mycorrhizal fungi (AMF, phylum Glomeromycota or subphylum Glomeromycotina; Spatafora et al. 2016) are a widespread group of plant root symbionts (Smith and Read 2008). AMF facilitate the uptake of water and mineral nutrients (e.g. phosphorus, nitrogen) in exchange for carbon compounds derived from plant photosynthesis (Parniske 2008; Rich et al. 2017) and are therefore widely studied as bioinoculants.

The effectiveness of AMF inoculation in agricultural fields has been demonstrated in several cases. For example, inoculation with *Funneliformis mosseae* and *Entrophospora etunicata* spores has been shown to improve drought tolerance in wheat (Al-Karaki et al. 2004), and inoculation with *Rhizophagus irregularis* spores increases phosphorus uptake in maize (Abrar et al. 2025). However, AMF inoculation does not always yield positive results. In some cases, it may fail due to incompatibility with local soil conditions or indigenous microbial communities (Kudla et al. 2021; Lutz et al. 2023). Additionally, exogenous AMF inocula can disrupt the diversity of native microbes and alter the functions of soil ecosystems (Trabelsi and Mhamdi 2013; Li et al. 2025). Indeed, AMF interact extensively with resident microbial communities, including bacteria and other fungi, in both positive and negative ways. Positive interactions include mutualism or facilitation, whereby AMF enhance the presence of plant growth-promoting rhizobacteria (PGPR) such as taxa related to the genera *Pseudomonas* and *Bacillus*. These further improve plant growth and stress resistance (Nanjundappa et al. 2019). Mycorrhiza-helper bacteria (MHB) such as *Acidobacterium*, *Brevibacillus* and *Arthrobacter* can stimulate root exudation or produce phytohormones themselves, inducing the germination of AMF spores and facilitating mycorrhizal colonization (Barea et al. 2005; Frey-Klett et al. 2007). Saprotrophic fungi such as *Trichoderma* spp. can enhance AMF root colonization, improving nutrient uptake and pathogen suppression (Harman et al. 2004; Mendoza-Mendoza et al. 2017).

However, AMF may experience antagonistic interactions with certain microbial groups due to competition for space and nutrients or even parasitism. For instance, *Pseudomonas putida* strain R104 has been reported to inhibit colonization by the AMF *R. clarus* NT4 by producing antimicrobial metabolites (Walley and Germida 1997), while *Fusarium* spp. may negatively impact AMF establishment and function (Cruz-Paredes et al. 2021). These competitive interactions may explain why locally sourced AMF inocula often perform better than introduced strains, as they are co-adapted to the resident microbiota (Rúa et al. 2016; Koziol and Bever 2016). Conversely, plant species significantly influence the composition and diversity of microbial communities in the root and rhizosphere, including AMF populations (Lei et al. 2018; Lepinay et al. 2024). Similarly, nitrogen-fixing bacteria such as *Rhizobium* are more prevalent in legumes than in cereal crops (Oldroyd et al. 2011). Despite this knowledge, the tripartite interactions between AMF, bacteria and other fungi remain poorly understood.

In addition to the host-specific interactions between certain plant species and their microbial communities, root exudates are another important driver shaping soil microbial diversity. These chemical compounds, such as sugars, amino acids, phenolics, organic acids and secondary metabolites, are essential in attracting and regulating beneficial microorganisms in the rhizosphere of various plant species (Trivedi et al. 2020). For example, benzoxazinoids, which are secreted by maize roots, can influence the composition of the rhizosphere microbiota and thereby the growth of bacteria that benefit plant health, such as *P. putida* (Kudjordjie et al. 2019). Furthermore, plant root exudates modulate interactions with AMF (Besserer et al. 2006), influencing fungal colonization and improving phosphorus and nitrogen acquisition (Garcia et al. 2016). However, variations in plant development and environmental conditions can lead to differences in exudate composition, affecting microbial recruitment and overall plant performance (Zhalnina et al. 2018). Thus, these root-derived compounds act as key mediators in shaping soil microbiota, reinforcing the beneficial interactions between plant and microbes that contribute to nutrient cycling and disease suppression.

Understanding these complex interactions is essential for designing optimal microbial consortia tailored to specific crops. Well-designed consortia that integrate AMF with beneficial bacteria and fungi can maximize plant health, improve nutritional efficiency and enhance tolerance to stressors (Bashan et al. 2014). Recent studies emphasize the importance of selecting compatible microbial partners to ensure the stability and efficiency of inocula under field conditions (Hart et al. 2018). In crops such as wheat, the world’s second-largest food source (Erenstein et al. 2022), tailored microbial communities can influence its growth, disease resistance and productivity. Beneficial microbial communities, including AMF, nitrogen-fixing bacteria (NFB), phosphate solubilizing bacteria (PSB) and biocontrol fungi (*Trichoderma*, *Gliocladium*), contribute to enhanced root growth, improved phosphorus solubilization and better nitrogen assimilation (Mahdi et al. 2010; Adedayo and Babalola 2023). Antagonistic microbes, such as *Bacillus subtilis*, can further suppress wheat pathogens like *Fusarium graminearum* and *Puccinia striiformis* f.sp. *tritici*, reducing the impact of diseases (Zhao et al. 2014; Reiss and Jørgensen 2017). A diverse and balanced rhizosphere microbiota is therefore essential to maintain wheat productivity under changing environmental conditions.

Thus, the aim of the present study was (i) to compare the wheat-associated microbiota (AMF, fungi and bacteria) from two ecological niches (roots and rhizosphere) with the microbiota of two mycotrophic plant species (clover and leek) and (ii) to analyze the co-occurrence networks of microbial communities in relation to the three plant species and their niches (roots vs. rhizosphere). The ultimate goal would be to identify the microbial communities associated with the three plant species, particularly those that interact positively with the AMF, for integration into optimized microbial consortia tailored to crops such as wheat.

## Materials and methods

### Soil sampling and pot experiment

Soil samples were collected on June 21, 2022, from an organically farmed wheat field (GAEC du Ransart, Frévillers) in the Hauts-de-France region. Three samples (A, B and C) (Online resource: SI Table 1) of wheat clods (wheat plants and the surrounding rhizosphere soil) were taken from a depth of 0 to 10 cm and placed in sterile bags. The soil was separated from the wheat roots by manual shaking and stored at approximately 7 °C. The physicochemical characteristics of the soil samples are provided in SI Table 2 (Online resource).

For a pot experiment, 250 mL of each soil sample (A, B and C) was mixed with 50 mL of perlite in each pot (10 cm diameter, 9 cm depth) (Online resource: SI Fig. 1). For each of the three soils (A, B and C), six pots were prepared, each with one of three plant species: two with wheat (*Triticum aestivum* cultivar Alixan), two with red clover (*Trifolium pratense* cultivar Chlumecky) and two with leek (*Allium porrum* cultivar Bleu de Solaise). This represents a total of 18 pots, corresponding to 6 pots for each plant species. The plants were grown in semi-controlled conditions at a 16:8 h day/night photoperiod in constant temperature (21 °C) and watered three times a week with distilled water to maintain soil water capacity. Throughout the experiment, the positions of the pots were randomized regularly to minimize the potential environmental impact (e.g. variations in light, air conditioning). After 8 months, the plants were removed from the pots, and the rhizospheric soil (the soil attached to the roots) was separated from the roots by washing with sterilized water and centrifugating for 5 min at 3,000 rpm to remove excess water. The samples were stored at -20 °C prior to further analyses.

### Total genomic DNA isolation

Total genomic DNA (gDNA) was extracted from the roots of three plant species and their respective rhizospheric soils. For each root sample, three replicates of 250 mg were weighed and ground in a mortar pre-treated with a 0.1% aqueous solution of diethyl pyrocarbonate water overnight and autoclaved twice to remove all traces of DNases and residues. The grinding was done using a pestle in liquid nitrogen, and 250 mg of the ground root tissue from each sample was used to extract genomic DNA using the “NucleoSpin Soil, Mini kit for DNA from soil” (Macherey-Nagel, Düren, Germany). Similarly, three replicates of 250 mg of rhizospheric soil were used to extract genomic DNA from the rhizosphere of the different plant species. DNA concentrations were measured using a Qubit Flex Fluometer™ (Invitrogen, Waltham, Massachusetts, USA) and standardized to 5 ng/µL^− 1^ with a final volume of 20 µL per sample for subsequent PCR.

### DNA amplification for AMF, bacterial and fungal communities

All the PCR were performed in triplicate. The AMF community was targeted using a nested PCR approach as described in Stefani et al. (2020). First, a 20-cycle PCR was performed to amplify the V3-V4-V5 regions (~ 795 bp) of the small subunit 18 S ribosomal RNA (SSU rRNA) using the primer pair AML1 (5′-ATCAACTTTCGATGGTAGGATAGA-3′) / AML2 (5′-GAACCCAAACACTTTGGTTTCC-3′) (Lee et al. 2008). The reaction mixture (20 µL) contained 1 µL of gDNA, 0.8 µM of each primer, 0.2 mM of each dNTP, 2 mM Mg²⁺ and 2.5 U of Q5 High-Fidelity DNA Polymerase (NEBNext^®^ Q5 Hot Start HiFi PCR Master Mix, New England Biolabs, Ipswich, MA, USA). DNA amplification was performed using a Biometra TProfessional thermocycler (Biometra GmbH, Göttingen, Germany). The thermocycling conditions consisted of an initial denaturation at 98 °C for 30 s, followed by 20 cycles of denaturation at 98 °C for 10 s, primer annealing at 64 °C for 30 s and extension at 65 °C for 60 s. A final extension step at 65 °C for 5 min was included, after which the samples were held at 4 °C. Positive controls (gDNA extract from *Rhizophagus irregularis*) and negative controls (sterilized water) were included in all reactions. To minimize the amplification of non-mycorrhizal sequences during the second-round PCR, first-round PCR products were purified using the QIAquick PCR Purification Kit (Qiagen, Valencia, CA, USA) according to the manufacturer’s instructions. Purified PCR products were then amplified using the primer set nu-SSU-0450-5′ (5′-CGCAAATTACCCAATCCC-3′) / nu-SSU-0899-3′ (5′-ATAAATCCAAGAATTTCACCTC-3′) (Online resource: SI Table 3). The amplification reaction mixture was similar to the first-round PCR, except for the primer concentration, which was 0.5 µM. The thermocycling conditions were the same as in the first-round PCR, except for the number of cycles, which was reduced to 15, and the annealing temperature, which was set to 59 °C.

The bacterial community was targeted by amplifying the hypervariable V3–V4 region of the 16 S rRNA gene using the primer set 515bF (5′-GTGYCAGCMGCCGCGGTAA-3′) and 926R (5′-CCGYCAATTYMTTTRAGTTT-3′). The fungal community was targeted by amplifying the ITS region using the primer set ITS-9 F (5′-GAACGCAGCRAAIIGYGA-3′) and ITS-4R (5′-CCTCCGCTTATTGATATGC-3′). PCR amplifications were performed from 1 ng of extracted DNA using a Surecycler 8800 thermal cycler (Agilent, Technologies, Les Ulis, France). The reaction mixtures (25 µL) contained 5 µL of 5× Q5 reaction buffer, 3 µL of 5× Q5 High GC enhancer (for fungal PCR), 0.25 µL (2 U µL^− 1^) of Q5^®^ High-Fidelity DNA Polymerase (New England Biolabs France, Evry, France), 0.8 µL of each primer (0.4 µM), 1 µL of dNTPs (0.2 mM), 1 µL of DMSO, 1 µL of Bovine Serum Albumin (BSA; 100 µg/mL^− 1^) and 1 ng of DNA template. For bacterial 16 S rRNA PCR, thermocycling conditions were as follows: initial denaturation at 95 °C for 3 min, followed by 35 cycles of denaturation at 95 °C for 30 s, primer annealing at 56 °C for 30 s and extension at 72 °C for 50 s, with a final extension at 72 °C for 5 min. For fungal ITS region PCR, thermocycling conditions were as follows: initial denaturation at 95 °C for 10 min, followed by 35 cycles of denaturation at 94 °C for 20 s, primer annealing at 47 °C for 30 s and extension at 72 °C for 20 s, with a final extension at 72 °C for 5 min.

All PCR products were visualized by electrophoresis on a 1.5% agarose gel stained with GelRed^®^ (1:10,000; Biotium Inc., Fremont, CA, USA) at 60 V for 40 min and visualized using the Gel-Doc system (Bio-Rad Laboratories, Mississauga, ON, Canada).

### MiSeq library preparation and sequencing

For AMF communities sequencing, library preparation followed the protocol described in Stefani et al. (2020). Briefly, nested PCR products were purified using Agencourt AMPure^®^ XP beads (Beckman Coulter Inc., Indianapolis, IN, USA), normalized to 1 to 2 ng/µL^− 1^ with the SequalPrep™ Normalization Plate kit (ThermoFisher Scientific) and indexed using the Nextera index kit (Illumina, San Diego, CA, USA). Indexed amplicons were then purified and normalized. Purified indexed amplicons were quantified by qPCR using the LightCycler^®^ 480 system (Roche Molecular Systems Inc., Branchburg, NJ, USA) with the KAPA library quantification kit for Illumina platforms (KAPA Biosystems, MA, USA) in order to determine the volume of each sample required to prepare a 1 nM amplicon library. Paired-end sequencing (2 × 300 bp) was carried out using the Illumina MiSeq^®^ sequencer (500 cycles) at the Molecular Technologies Laboratory of the Ottawa Research and Development Centre (Agriculture and Agri-Food Canada).

The PCR products representing the fungal and bacterial communities (18 rhizosphere samples and 18 root samples) were pooled and sequenced at the Genome Québec Innovation Centre (Montréal, QC, Canada) using Illumina MiSeq paired-end (2 × 300 bp) sequencing.

### Bioinformatic analyses

Bioinformatic analyses were performed using QIIME2 (v.2024.5) (Bolyen et al. 2019). Sequence denoising was carried out with DADA2 plugin. Sequences were trimmed to include only bases with quality scores > 35. The first 21 and 24 nucleotides from the 5′ end of the forward and reverse sequences, respectively, were removed. The 3′ ends of the forward and reverse sequences were truncated at positions 247 and 243, respectively. Amplicon sequence variants (ASVs) with a frequency of less than 0.1% of the mean sample depth were removed. For bacteria, ASVs were used directly for subsequent diversity analyses, whereas for the AMF and fungal ITS datasets, de novo OTU clustering was performed at 100% and 97% of similarity thresholds, respectively, using VSEARCH from QIIME2 (Rognes et al. 2016). Taxonomic assignment of the representative sequences was performed using SILVA (Quast et al. 2013) for AMF and bacterial communities and UNITE (Abarenkov et al. 2010) for total fungal communities. Alpha- and beta-diversity metrics were calculated from ASV/OTU tables using the microeco R package v1.9.1 (Liu et al. 2021). Rarefaction was performed at 20,000, 9000 and 10,000 sequences per sample for 18 S-AMF, 16 S-bacterial and ITS-fungal datasets, respectively. Shannon and Chao1 indices, as well as Hill numbers (q = 0, q = 1 and q = 2), were calculated as indicators of microbial alpha-diversity, while microbial beta-diversity was assessed using principal coordinate analysis (PCoA) based on Bray-Curtis distances matrices.

### Statistical and network analyses

Data were analysed using R v4.3.1 (R Core Team 2023). For all univariate data, the assumptions of normality and homoscedasticity were checked using the Shapiro-Wilk and Levene’s tests, respectively. The impact of different factors (niche and plant species) on AMF, fungal and bacterial alpha-diversity was tested by ANOVAs, followed by Tukey’s pairwise tests. The effect of plant species on microbial composition was analyzed at different taxonomic levels (phylum and genus) using linear discriminant analysis (LDA) with the *LefSE* R package (Segata et al. 2011).

Interkingdom networks were analyzed using the microeco R package. Sequences belonging to the *Glomeromycotina* subphylum were removed from the ITS dataset, because only the 18 S sequences were considered for the AMF. The three ASV/OTU tables were combined to calculate co-occurrences between the representative sequences. ASVs/OTUs representing less than 0.01% of the total ASV abundance were removed. For each soil niche, the Spearman correlation test was used to test for co-occurrence between ASVs/OTUs for each plant species with a *p*-value threshold of *p* < 0.05. An association was considered significant when *r* > 0.7 or *r* < -0.7. Then, co-occurrence networks were constructed for each plant species using the *igraph* R package, and module partitioning was performed using the *cluster_fast_greedy* algorithm. Network properties (number of nodes and edges, proportion of positive/negative edges, average degree) were compared among plant species in the two soil niches. Network visualization was performed using Gephi v0.10.1 (Bastian et al. 2009).

## Results

### Niches and plant identity drive microbiota diversity

**Fig. 1:**
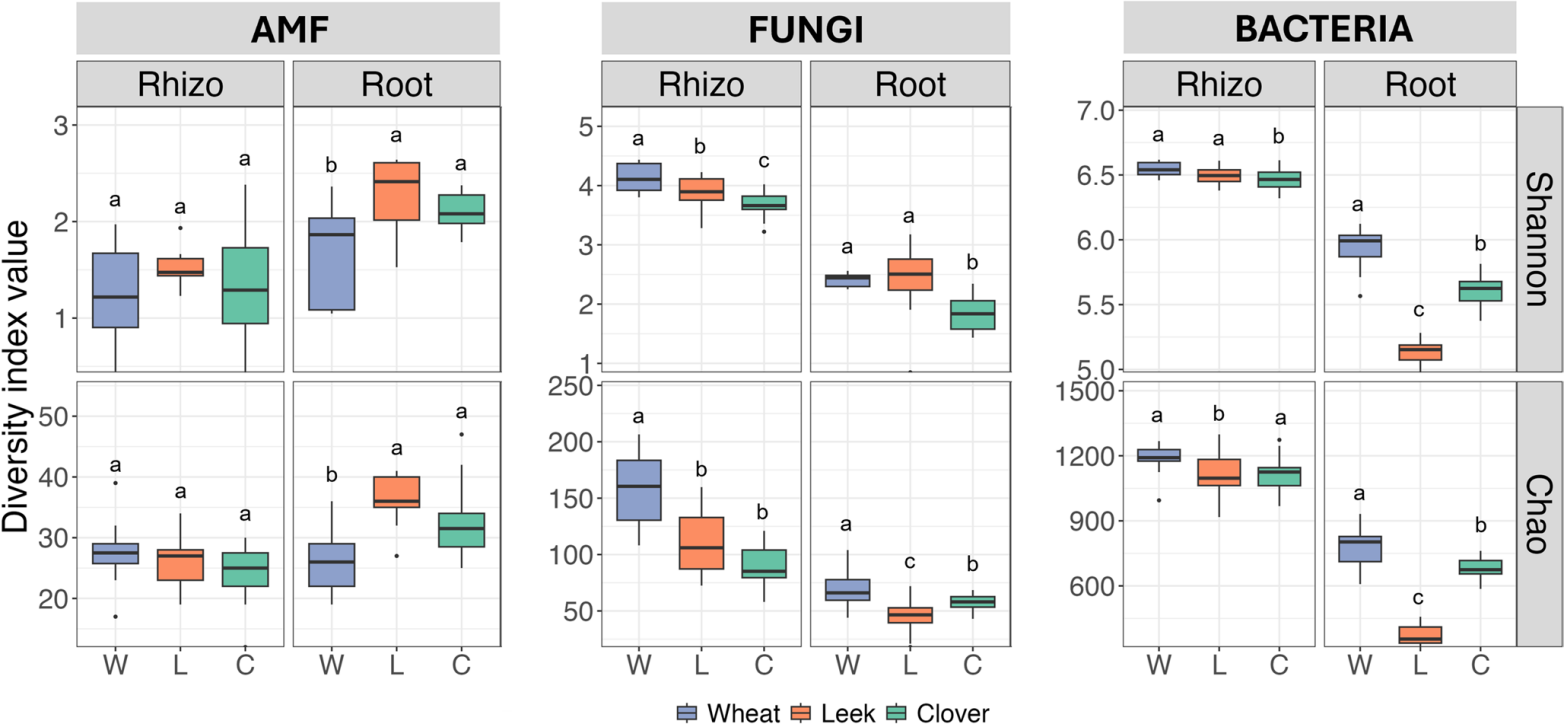
Alpha diversity of AMF, fungi and bacteria in the rhizosphere and roots of wheat (W), leek (L) and clover (C). Boxplots show the distribution of Shannon (top panels) and Chao (bottom panels) diversity indices for each plant–niche combination. In each boxplot, the central line represents the median, the box spans the interquartile range (IQR, 25th–75th percentile) and the whiskers extend to 1.5 × IQR; points beyond the whiskers indicate outliers. Y-axes are scaled independently in each panel to enhance the visibility of differences among treatments. Different letters above the boxplots indicate significant differences between plant species, as determined by ANOVA followed by Duncan’s test (α = 0.05)

Alpha-diversity analysis based on Chao1 index, Shannon index (Fig. 1) and Hill numbers (q0: Species richness, q1: exponential Shannon entropy and q2: inverse Simpson index) (Online resource: SI Fig. 2) revealed similar patterns of microbial diversities across niches and the three tested plant species (wheat, clover, leek). In the rhizosphere, the richness and diversity of AMF (Chao1 and Shannon indices, respectively) did not differ significantly across all plant species. However, AMF alpha-diversity was significantly lower in wheat roots (Chao1 index: 26.56; Shannon index: 1.68; q0: 26; q1: 6.45) than in leek and clover roots (Chao1 index: 36.38 and 32.86; Shannon index: 2.25 and 2.1; q0: 36 and 31.5; q1: 11.17 and 8.01). A similar trend was observed for q2, suggesting that dominant AMF taxa were also less diverse in wheat roots compared to those of the other plant species. Wheat had higher fungal communities’ richness (Chao1 index: 115.14, 4.12; q0: 158, 63.5) than leek (Chao1 index: 75.06, 45.19; q0: 105, 46.5) and clover (Chao1 index: 73.6, 57.86; q0: 85, 57.5) in both the rhizosphere and roots. A similar pattern was observed for bacteria: wheat showed the richest and most diverse communities in rhizosphere and roots (Chao1 index: 954.71, 780.33; Shannon index: 6.2, 5.94; q0: 1141, 773.5; q1: 680.75, 400.19; q2: 376.25, 196.36), followed by clover (Chao1 index: 895.8, 680.75; Shannon index: 6.04, 5.61; q0: 1107, 354; q1: 667.36, 170.88; q2: 380.01, 83.37) and leek (Chao1 index: 727.73, 375.42; Shannon index: 5.79, 5.14; q0: 1069, 650; q1: 667.36, 172.88; q2: 333.92, 120.15). Compared to wheat rhizosphere, q2 index indicates that wheat roots host a higher number of dominant taxa compared with the other plant species.

**Fig. 2:**
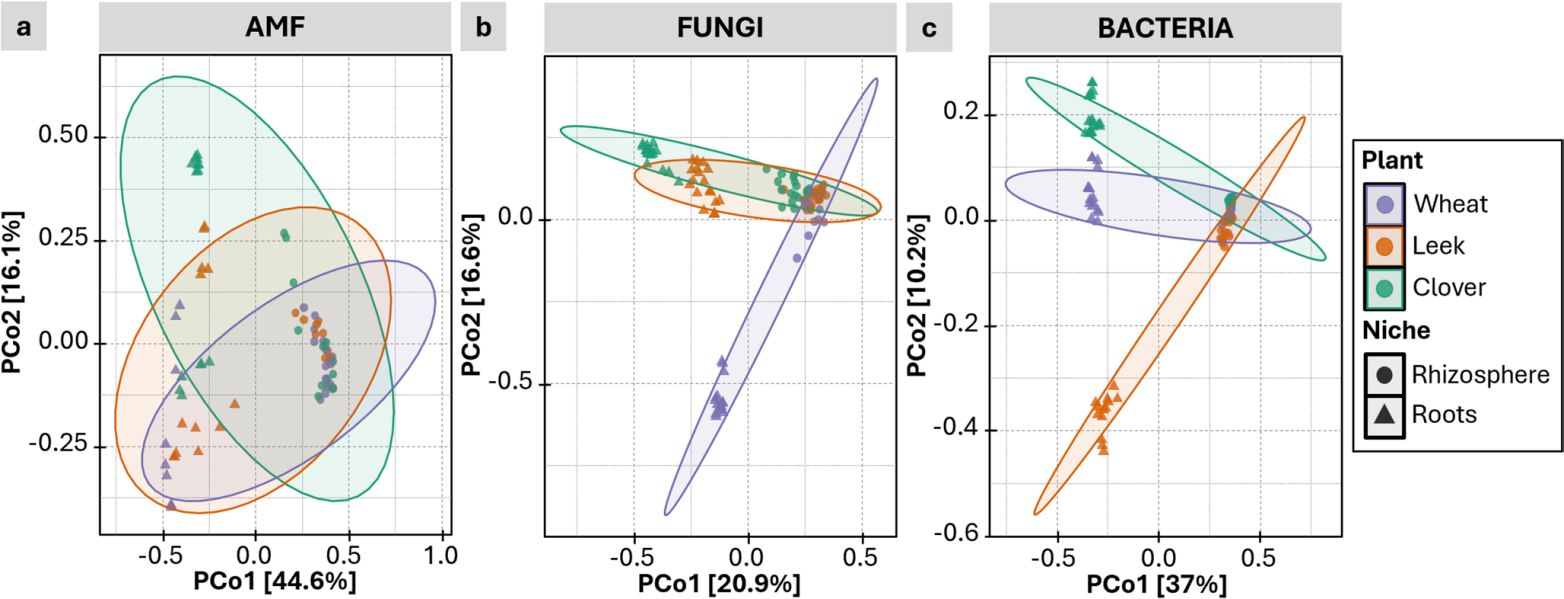
Principal component analysis (PCoA) based on Bray-Curtis distance metrics of (**a**) arbuscular mycorrhizal fungi (AMF), (**b**) fungal and (**c**) bacterial communities, according to plant species and niches (rhizosphere and roots)

Beta-diversity analyses of AMF, fungi and bacteria showed a clear separation between the rhizosphere and root niches, which explained most of the variance (Fig. 2). However, the host plant had different impacts on the microbial communities. For AMF, the communities observed in wheat, leek and clover overlapped strongly, indicating the community was weakly structured by plant identity (Fig. 2a). Plant identity had a much stronger effect on fungal and bacterial communities, especially in roots (Fig. 2b and c). The fungal and bacterial communities recovered in wheat and leek roots, respectively, formed distinct clusters.

**Fig. 3:**
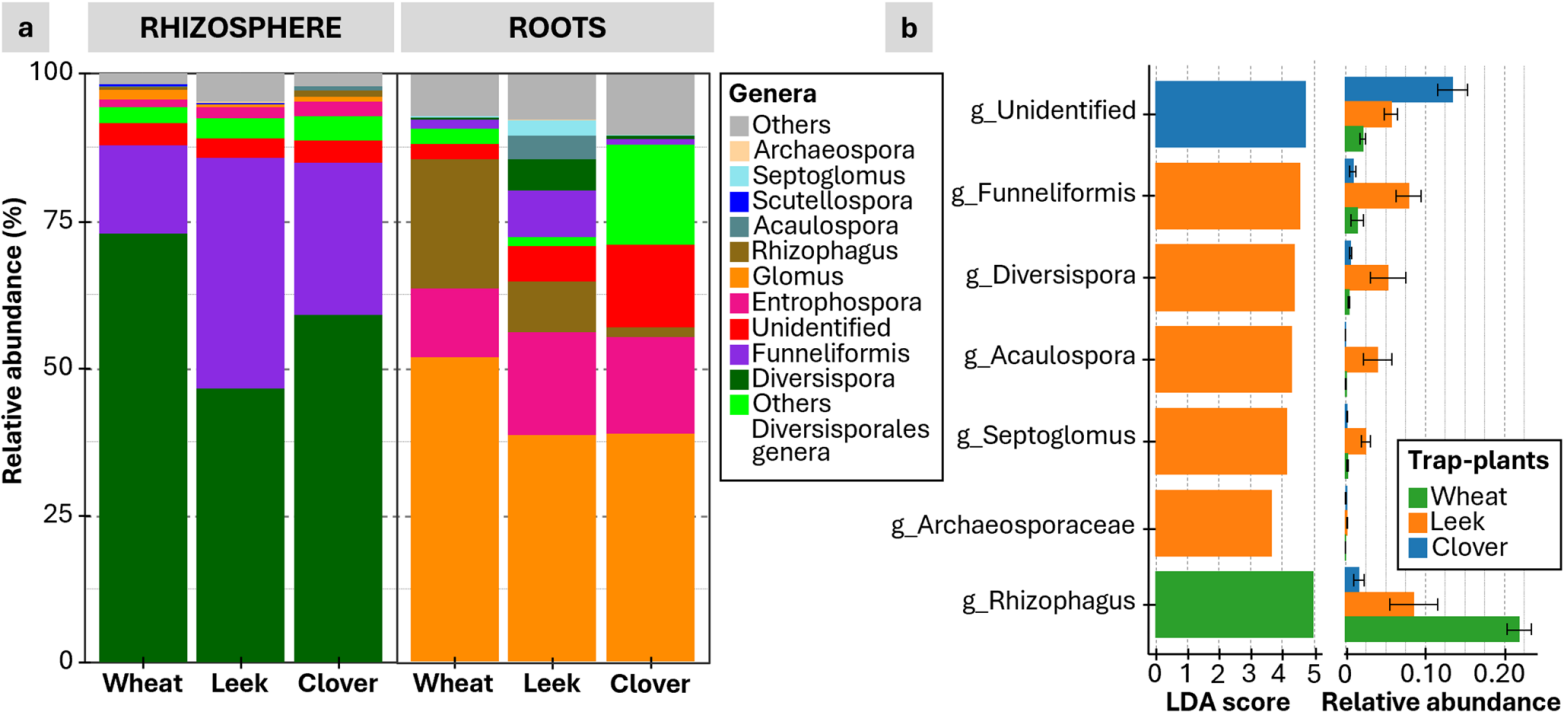
AMF composition in the roots and rhizospheres of the three trap plant species. (**a**) Relative abundances of dominant *Glomeromycota*, (**b**) Linear discriminant effect size (left) plot showing AMF genera that are differentially abundant across the rhizospheres of the three plant species. The relative abundances of these genera are displayed on the right side of the LDA score bars

Analyses of the relative taxonomic abundance confirmed that the niche had a stronger, though still plant-dependent, effect on the microbial communities. For AMF, the communities recorded in the rhizosphere of all three plants were dominated by *Diversispora* (45% to 70%) and *Funneliformis* (14% to 38%). The wheat rhizosphere was richer in *Diversispora*, the leek rhizosphere was richer in *Funneliformis* and the clover rhizosphere was intermediate (Fig. 3). The composition of AMF shifted markedly in roots: several genera became enriched, especially *Glomus*, which dominated wheat roots (45% to 70%), and *Rhizophagus*, which was more abundant in wheat and, to a lesser extent, in leek roots (Fig. 3a). Clover roots showed a higher contribution of taxa related to the order Diversisporales (Fig. 3a). Leek roots retained comparatively high proportions of both *Diversispora* and *Funneliformis*, whereas these genera declined in wheat and clover roots. AMF genera differed significantly among plant rhizospheres (Fig. 3b). *LefSe* R package identified distinct indicator genera for wheat, leek and clover, which is consistent with the host-driven shifts in community composition observed in Fig. 3a.

**Fig. 4:**
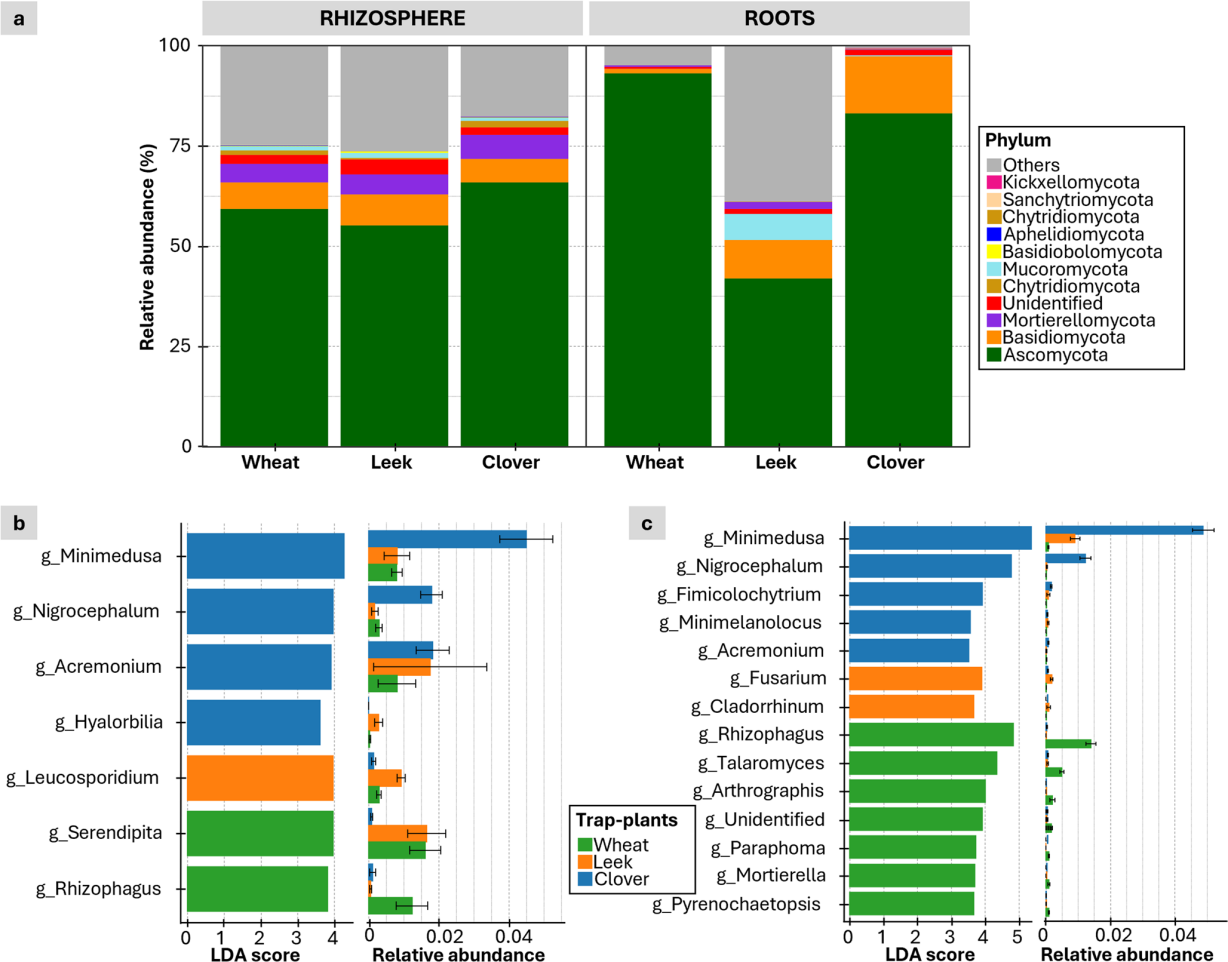
Total fungal composition in the roots and rhizospheres of the three trap plant species. (**a**) Relative abundances of fungal phyla in the rhizosphere and the root. Linear discriminant analysis (LDA) effect size plots show specific fungal genera that are differentially abundant across the three plant species in the rhizospheres (**b**) and roots (**c**). The relative abundances of the corresponding genera are displayed to the right of the LDA score bars

For fungi, the phylum-level composition of rhizosphere communities was broadly similar among the three plants, except for Ascomycota, which was more abundant in clover (Fig. 4a and c). In contrast, root communities differed significantly. Mucoromycota (which includes AMF), were enriched in leek roots. Basidiomycota were more abundant in clover and leek roots (14% and 9%, respectively) than in wheat roots. Ascomycota were twice as abundant in clover and wheat roots as in leek roots (Fig. 4a). At the genus level, LDA identified *Rhizophagus* as being strongly associated with wheat in both rhizosphere (15- and 9-fold higher) and root samples (38- and 21-fold higher; Fig. 4b and c). *Minimedusa* and *Nigrocephalum* were characteristic of clover and were much more abundant in its rhizosphere and roots than in wheat (5-fold higher in rhizosphere and 43- and 35-fold higher in roots) or leek (5- and 9- fold higher in rhizosphere and 5- and 19-fold higher in roots; Fig. 4b and c).

**Fig. 5:**
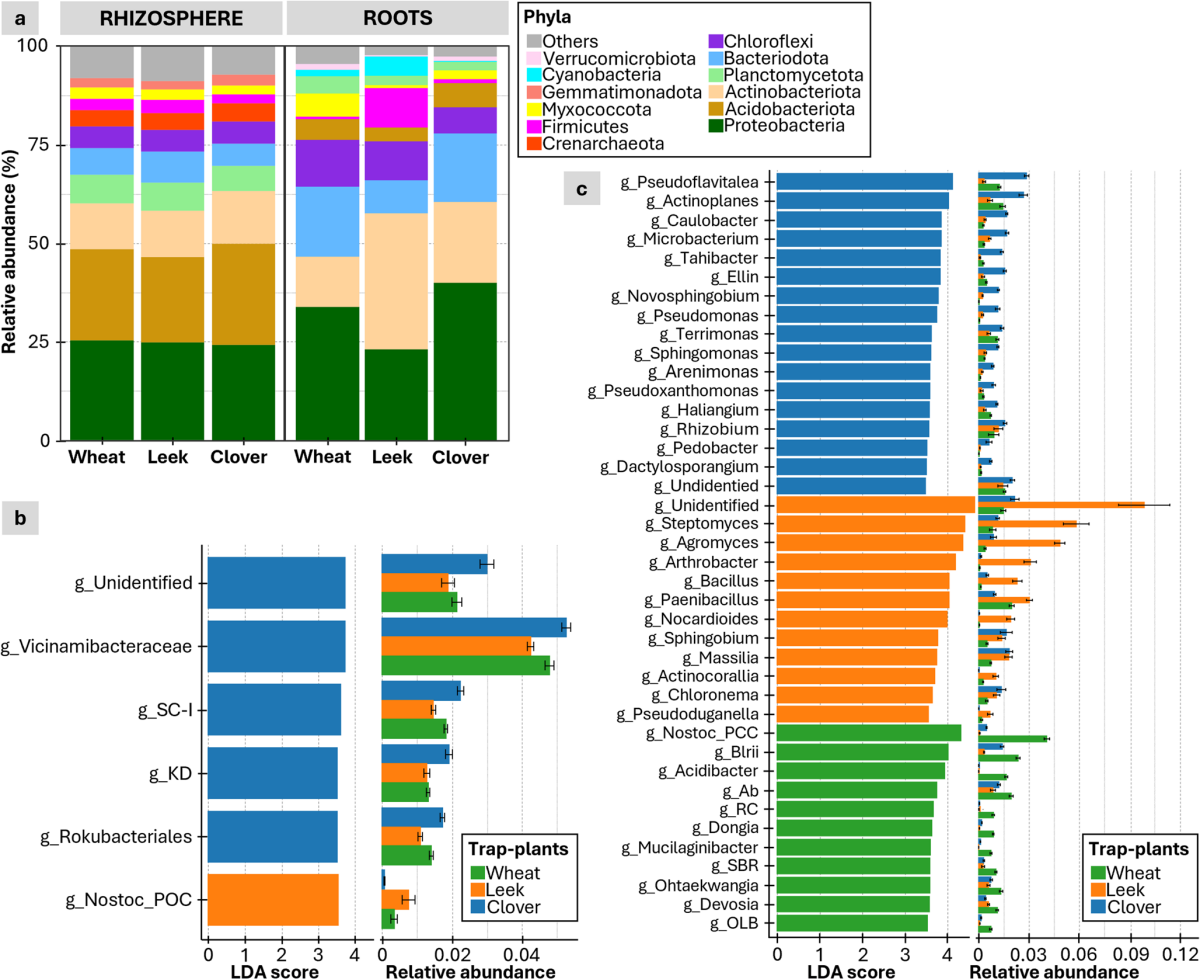
Bacterial composition in the roots and rhizosphere of three trap plant species. (**a**) Relative abundance of dominant bacterial phyla. Linear discriminant analysis (LDA) effect size graph (left) shows bacterial genera that are differentially abundant across the rhizospheres of the three plant species in the rhizosphere (**b**) and the roots (**c**). The relative abundances of the corresponding genera are displayed on the right of the LDA score bars

For bacteria, plant identity had a stronger effect on bacterial composition in roots than in the rhizosphere. Similar phylum-level profiles were observed in the rhizosphere of the three plants, dominated by Acidobacteriota, Proteobacteria and Actinobacteriota (Fig. 5a). In contrast, root communities differed greatly depending on the host plant. Proteobacteria and Bacteroidota were enriched in wheat and clover, Chloroflexi were enriched in wheat and leek, and Actinobacteriota were enriched in clover and leek (20.5% and 34.6%, respectively). Firmicutes were particularly abundant in leek (10%) compared to clover and wheat (0.97% and 5.9%, respectively). LDA showed a few host-specific genera in rhizosphere, but several in root samples, especially in leek roots (Fig. 5b and c). Genera such as *Streptomyces*, *Agromyces*, *Arthrobacter*, *Bacillus* and *Paenibacillus* were strongly enriched in leek roots compared to those of wheat and clover (Fig. 5c).

### Plant identity shapes cross-kingdom interaction networks

**Fig. 6:**
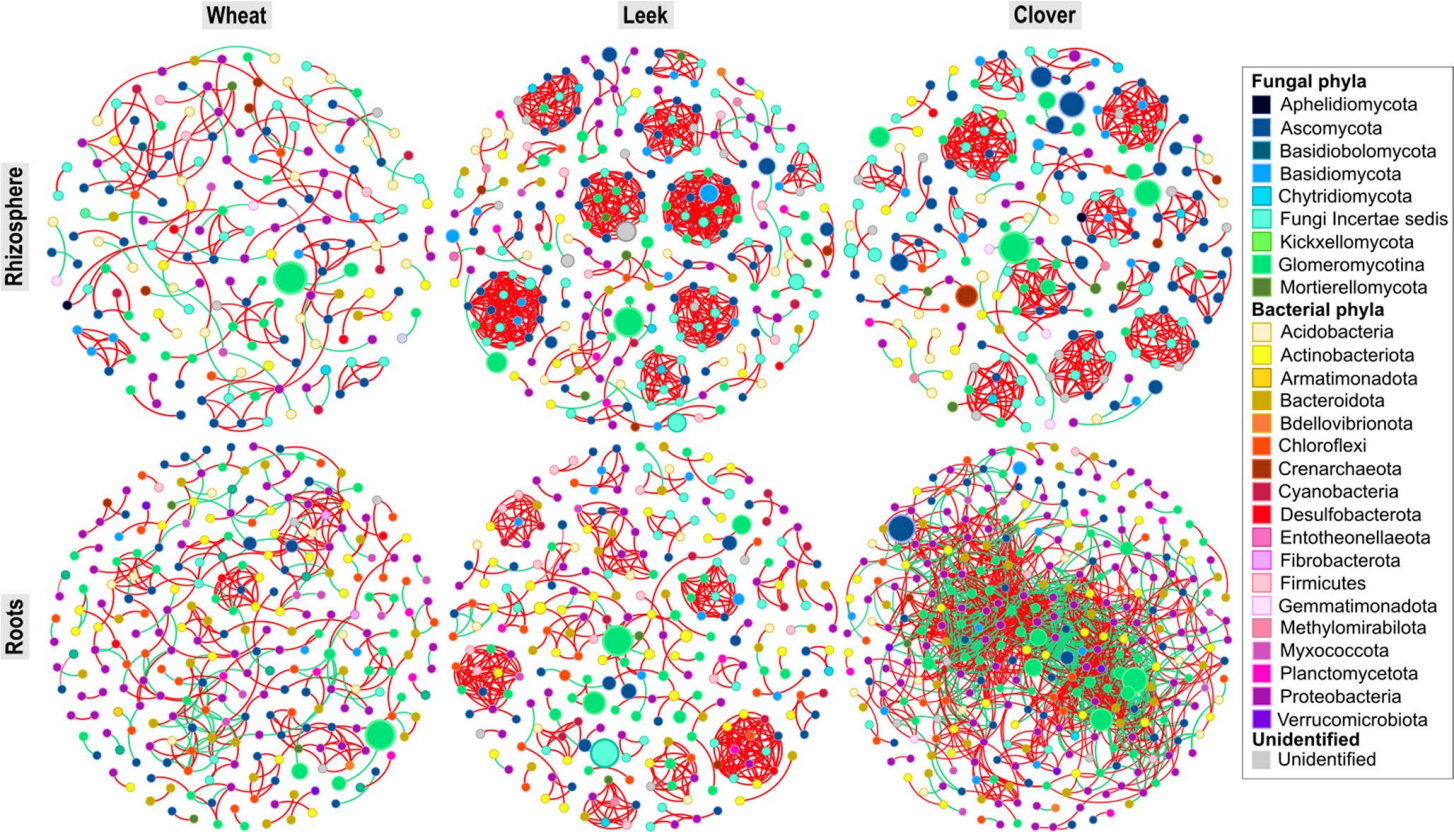
Co-occurrence networks illustrating positive and negative correlations between AMF ASVs, fungal OTUs (excluding *Glomeromycotina*) and bacterial ASVs, based on niche and plant species. Each circle or node represents an OTU or ASV that is significantly associated (Spearman correlations *p* < 0.05, *r* > 0.7) with one or more OTUs/ASVs. Lines indicate positive correlations (red) and negative correlations (green). The correlation threshold was set at 0.7 according to RMT theory (Deng et al. 2012)

Microbial co-occurrence networks were less connected in wheat than in the two mycotrophic plants, particularly in the rhizosphere. Leek and clover had more nodes and edges, as well as a higher proportion of positive links (Fig. 6; Table 1).

In the rhizosphere, wheat had the sparsest network, with fewer nodes (181) and edges (171), while leek and clover showed much denser, highly connected networks (Fig. 6; Table 1). Most interactions were positive in all three plants, but the proportion of positive edges was clearly lower in wheat (81.8% of all interactions) than in leek and clover (96.7% and 96.5%, respectively). Rhizosphere networks were dominated by fungal-fungal and fungal-bacterial interactions, particularly in leek and clover. For instance, fungal-fungal interaction in leek rhizosphere accounted for 87.1% of all edges. For AMF, more than 90% of interactions were positive across plant species and occurred predominantly with other fungi (76.7% to 81.6% of AMF edges).

**Table 1:** Number of modules, nodes and edges according to niches and trap plant species

		Rhizosphere						Roots					
		Wheat		Leek		Clover		Wheat		Leek		Clover	
Module number		52		73		63		56		68		34	
Node number		181		266		218		263		258		339	
Edge number		171		687		459		359		454		1530	
Edge types		+	-	+	-	+	-	+	-	+	-	+	-
% edges	AMF–AMF	3.5	0.6	2.9	0.0	3.9	0.0	3.1	0.3	2.9	0.0	4.6	2.7
	AMF–NGF	13.5	0.0	19.4	0.0	23.1	0.2	3.9	1.1	6.2	0.4	5.2	2.7
	AMF–Bacteria	0.6	1.2	2.2	0.1	1.3	0.4	9.7	1.9	11.2	0.4	18.4	11.6
	NGF–NGF	31.0	0.6	64.8	0.0	63.0	0.0	3.6	1.1	8.4	0.0	2.3	0.5
	NGF–Bacteria	15.2	3.5	2.9	0.4	2.6	0.9	17.0	7.2	27.3	1.3	11.4	5.3
	Bacteria–Bacteria	18.1	12.3	4.5	2.8	2.6	2.0	36.5	14.5	39.2	2.6	21.8	13.6

The percentages of each type of interaction between microbial communities (AMF to AMF; AMF to non-*Glomeromycotina* fungi (NGF); AMF to bacteria; NGF to NGF; NGF to bacteria; bacteria to bacteria), whether they are positive or negative, are given for all interactions for each trap plant in each niche (rhizosphere and roots)

In the roots, the network structure shifted and became more bacteria driven (Fig. 6; Table 1). Network complexity increased from the rhizosphere to the roots in wheat (263 nodes and 359 edges) and especially in clover, which exhibited the most complex and densely connected root network (339 nodes and 1530 edges). Leek roots showed a slight reduction in connectivity (258 nodes and 454 edges) compared to the rhizosphere (266 nodes and 687 edges). In all three plants, most edges involved bacterial-bacterial and bacterial-fungal interactions (> 82% of all edges). AMF-bacterial interactions were rare in the rhizosphere but became much more frequent in the roots, particularly in clover, where they represent 30% of all edges.

**Fig. 7:**
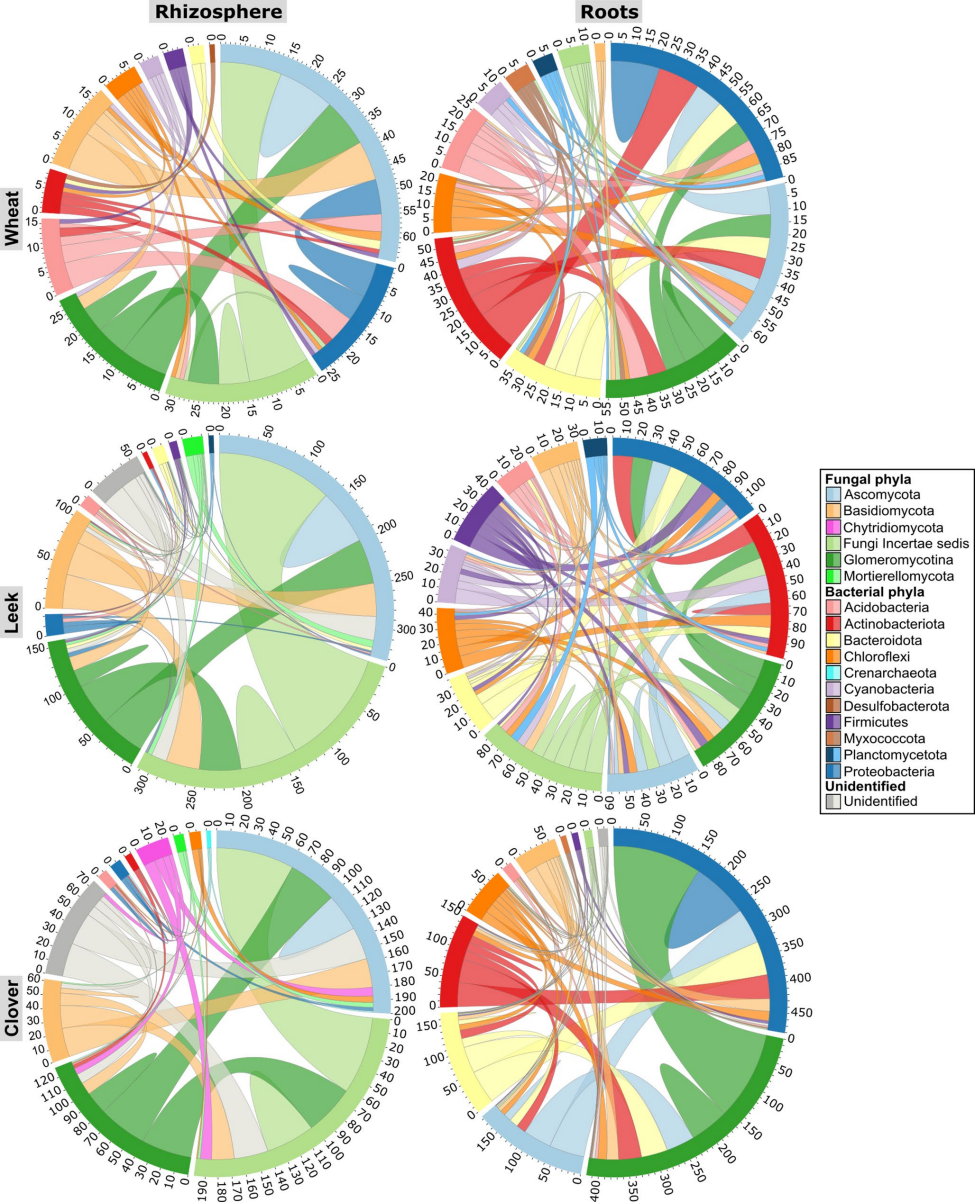
Chord diagrams representing the interactions (positive and negative interactions are not discriminated) between the different phyla of microbial communities (AMF; *non*-*Glomeromycotina* fungi; bacteria) according to niches and trap plant species

Interactions among phyla (nodes or chords colours) (Figs. 6 and 7) highlighted the primary AMF partners. In the wheat rhizosphere, only 23 positive interactions were detected between AMF and other fungi, primarily with Ascomycota (Fig. 7 and Online resource: SI Table 5). By contrast, AMF interacted with more fungi in the leek (133 positive interactions) and clover (106 positive and one negative interactions) rhizospheres, most of them being saprophytic fungi (Fig. 7 and Online resource: SI Table 6). Some were shared across all plant species (e.g. *Serendipita* sp.), while others were shared only between specific hosts (e.g. *Fusidium* sp. and *Cladosporium* sp. in wheat and clover and *Neoschizothecium* sp. in wheat and leek; Online resource: SI Table 5).

In the root niche, there were fewer AMF-bacteria interactions (35 positive and 7 negative interactions) in wheat than in leek (51 positive and 2 negative interactions) and clover (282 positive and 177 negative interactions), involving mainly Acidobacteria and Proteobacteria (Fig. 7 and Online resource: SI Table 7). In leek and clover, however, AMF interacted primarily with Actinobacteriota and Proteobacteria. There were also strong associations with Bacteroidota and Chloroflexi in clover (Fig. 7 and Online resource: SI Table 7). These bacterial partners were primarily saprophytes, plant growth-promoting rhizobacteria (PGPR) and AMF endosymbionts (Online resource: SI Table 8). Some genera, such as *Pseudonocardia* were shared between wheat and leek roots, while *Gaiella*, *Novosphingobium*, *Pseudomonas*, *Streptomyces* and *Terrimonas* were common AMF partners in leek and clover roots. Overall, AMF from the genera *Glomus*, *Rhizophagus* and *Entrophospora*, as well as genera within the order Diversisporales, interacted most positively with other fungi and bacteria across all plant species in both niches.

## Discussion

The present study aims to identify candidate AMF, fungal and bacterial taxa and their positive interactions in the rhizosphere and roots of wheat, leek and clover to improve the design of AMF-based inocula. The rhizosphere and root microbiota associated with wheat differ from those of leek and clover in diversity and co-occurrence interactions. Co-occurrence network data can provide a useful framework for designing microbial consortia to support plant health and nutrition.

### Effects of niches and crop species on microbial diversity

The same dominant bacterial and fungal phyla were detected in all three plant species. In the rhizosphere and roots, AMF communities were dominated by Glomerales, which include species typically found in high-input agroecosystems where intensive management acts as a selective filter (Oehl et al. 2003; Brito et al. 2012; Moebius-Clune et al. 2013). However, bacterial and fungal alpha-diversity were generally lower in the roots than in the rhizosphere. PCoA revealed strong, niche-driven shifts in community structure, as reported previously for pearl millet (Alahmad et al. 2023). Root exudates of photosynthates enrich the rhizosphere and attract microorganisms (Bais et al. 2006). Then, the physical boundary of rhizoplane and microorganism’s lifestyle impose a strong selection (Lugtenberg and Kamilova 2009; Bulgarelli et al. 2013). AMF genera also showed clear niche preferences: *Diversispora* and *Funneliformis* were more abundant in the rhizosphere, while *Glomus*, *Entrophospora* and *Rhizophagus* dominated in the roots. This pattern is consistent with contrasting life history strategies (Hart et al. 2001; Chagnon et al. 2013). *Glomus*, *Rhizophagus* and *Entrophospora* mainly develop their structures inside plant roots and grow rapidly (de Souza et al. 2005), whereas *Diversispora* relies more on host carbon and develops extensive extraradical hyphae, a K-strategy trait favouring rhizosphere dominance (Yan et al. 2025). LDA further highlighted plant-specific enrichment of bacterial and fungal species. Consistent with previous studies (Sellstedt and Richau 2013; Yousuf et al. 2017; Liu et al. 2019), leek roots were enriched with PGPR-type bacteria, including N-fixing Firmicutes (e.g. *Bacillus*, *Paenibacillus*) and Actinobacteria (e.g. *Streptomyces*, *Agromyces*, *Arthrobacter*). Clover roots were dominated by Proteobacteria (e.g. *Pseudomonas*, *Sphingomonas*, *Arenimonas*, *Pseudoxanthomonas*, *Rhizobium*). This is consistent with clover’s known symbiotic relationships with N-fixing rhizobia (Chen et al. 2003; MacLean et al. 2007). However, wheat did not show strong enrichment of particular PGPR but harboured the highest abundance and diversity of Ascomycota, including *Talaromyces*, *Arthrographis*, *Paraphoma* and *Pyrenochaetopsis*. These results suggest that wheat-associated bacterial and fungal communities are less host-specific and more diverse than those of mycotrophic species. AMF richness and diversity were lower in wheat roots than in clover and leek roots. Modern wheat cultivars, such as Alixan, may rely less on AMF because breeding has increased their intrinsic capacity to acquire phosphorus and nitrogen (Calderini et al. 1995; Hetrick et al. 2011; Cormier et al. 2016). On the contrary, leek and clover are strongly mycotrophic and are more dependent on AMF to acquire phosphorus and nitrogen (Tawaraya 2003; Yoneyama et al. 2008; Fang et al. 2020), which likely explains their more diverse AMF communities.

### Niches and plant species effects on interactions between AMF, fungi and bacteria

Co-occurrence networks showed a clear niche contrast. In the rhizosphere, interactions were largely driven by fungi. This could be related to the formation of mycelial networks by non-*Glomeromycotina* fungi, which benefit from the carbon coming from root exudates and contribute to decomposition and nutrient cycling (Yuan et al. 2021). In contrast, most interactions in roots were driven by bacteria, which can more easily enter root tissues through wounds, young roots and mycorrhizal hyphae (Sturz et al. 2000; Gaiero et al. 2013; Overbeek and Saikkonen 2016). Inside the roots, bacteria benefit from a continuous carbon supply (Waller et al. 2020).

Network structure also varied among the three plant species, indicating that the plant species could also play a role in structuring microbial interactions in both niches. Despite its high bacterial and fungal richness, wheat showed fewer edges and clusters than leek and clover, particularly in the rhizosphere. As explained by Yu et al. (2019), high microbial diversity can limit effective interactions due to competition and functional redundancy. Compared with wheat, leek and clover harboured specific PGPR-type and NFB bacterial diversity, and interactions among these bacteria were more pronounced in these plants. Likewise, positive interactions between AMF and other microorganisms were often involved different microbial genera depending on the plant species (Online resources: SI Tables 5, 6, 7 and 8). The three plant species used in this study are phylogenetically distant (Zuntini et al. 2024). Bread wheat and leek are monocotyledon plants from Poaceae and Liliaceae families, respectively, and clover is a dicotyledon of the Fabaceae family. Even under identical soil conditions, studies have demonstrated that plant species distinctively shape the diversity and abundance of microbial communities within the rhizosphere (Ofek-Lalzar et al. 2014) and roots (Edwards et al. 2015), leading to divergent microbe-microbe interactions. Specifically, monocotyledonous and dicotyledonous plants exhibit contrasting root morphologies, such as the prevalence of lateral and seminal roots in monocots, and distinct cellular metabolisms. These physiological differences can drive unique microbiome compositions and alter the nature of interactions between root-inhabiting microorganisms (Yu and Hochholdinger 2018; Wang and Sugiyama 2020; Kannaiah et al. 2025). Moreover, root exudates are known to modulate the rhizosphere environment by suppressing pathogens and selectively recruiting specific microbial taxa (Sasse et al. 2018), which ultimately drives the structure of microbial interactions (Zhao et al. 2021). For instance, plant species from Poaceae family (e.g. wheat, maize) produce Benzoxazinoids molecules which act as key modulators of root-associated microbiomes by selectively enriching or suppressing specific microbial taxa and reshaping microbial co-occurrence networks, thereby influencing inter-microbial interactions in both the rhizosphere and endosphere (Kudjordjie et al. 2019). Roots of *Allium* species are known to exudate dimethyl disulfide (DMDS) (Afridi et al. 2024). Some studies have reported that DMDS applications can have a nematocidal (Afridi et al. 2024) and fungicidal (Tyc et al. 2015) effects, or can attract or stimulate specific bacteria development (Garbeva et al. 2014; Schulz-Bohm et al. 2018). Fabaceae roots secrete isoflavonoids, inducing symbiosis with NFB *Rhizobia* (Santoyo 2022), which can themselves interact with other endophytes with the aim of improving root nodulation and plant fitness (Youseif et al. 2021). Despite these considerations, Fang et al. (2024) pointed out that the role of plant species, particularly their specific root exudates, in modulating cross-kingdom microbial interactions remains unclear and warrants further investigation.

Across all plants, AMF formed mostly positive co-occurrences, interacting preferentially with fungi in the rhizosphere and with bacteria in the roots. AMF transfer between 5% and 20% of host photosynthates to the soil (Jakobsen and Rosendahl 1990; Pearson and Jakobsen 1993), establishing a distinct hyphosphere that provides microorganisms with carbon and habitat (Andrade et al. 1997; Emmett et al. 2021; Zhang et al. 2022). Here, AMF were primarily associated with Proteobacteria (e.g., *Rhizobium*, *Caulobacter*, *Duganella*, *Lysobacter*, *Massilia*, *Pseudomonas*, *Tahibacter*) and Actinobacteria (e.g., *Actinoplanes*, *Agromyces*, *Gaiella*, *Pseudonocardia*, *Streptomyces*), many of which are PGPR, PSB or NFB, enhancing N and P availability. For example, NFB can have higher urease activity in the presence of AMF, increasing the availability of mineral nitrogen for plants (Kong et al. 2018). AMF can also induce the transcription of alkaline phosphatase genes in PSB through carbon signals, such as fructose (Zhang et al. 2018). MHB that interact with AMF (e.g. *Rhizobium*) can stimulate root exudation and produce phytohormones, inducing spore germination and host colonization (Frey-Klett et al. 2007). Many of these bacteria are found in the hyphosphere or are AMF endosymbionts such as *Bacillus*, *Haliangium* and *Massilia* (Ujvári et al. 2024). Hyphosphere-associated bacteria, including PGPR and antagonists such as *Actinoplanes*, can travel through hyphae into plant roots (de Novais et al. 2020; Ujvári et al. 2024). This facilitates their establishment in plant tissues and amplifies the positive effects of mycorrhization (Hnini et al. 2024). Here, AMF co-occurred also with many saprotrophic fungi such as *Trichoderma*, *Cladosporium*, *Acremonium*, *Mortierella* and *Enterocarpus*. Saprotrophic fungi co-inoculated with AMF, such as *Trichoderma* or *Mortierella*, can enhance crop growth in plants like melon (*Cucumis melo*) and Virginia saltmarsh mallow (*Kostelelzkya virginica*) (Martínez-Medina et al. 2011; Zhang et al. 2011). The Basidiomycota genus *Serendipita*, which interacted with AMF in clover and leek roots, includes numerous endophytic species that improve plant nitrogen uptake (Hallasgo et al. 2020; Bertolazi et al. 2025).

### Designing AMF-based microbial inocula from co-occurrence networks

Co-occurrence analyses provide a framework for assembling multi-partner, more effective microbial inocula as proposed by Shayanthan et al. (2022). Microorganisms that co-occur positively with AMF and benefit plants are logical candidates to create generalist or crop-specific consortia. Co-occurrence networks indicate that suitable partners are root-endophytic bacteria (NFB, PSB, PGPR and AMF-endosymbionts) and other soil fungi that colonize roots or mineralize organic matter in the rhizosphere. A generalist inoculum could include taxa that interact with AMF in both non-mycotrophic plants, such as wheat, and mycotrophic plants, such as leek and clover. Examples of these taxa include *Serendipita*, *Tahibacter*, *Pseudonocardia* and *Fusidium*. An inoculum targeted to wheat should include the few taxa that interact with AMF in the wheat rhizosphere, such as *Cephalotrichum*, *Fusicolla*, *Lasionectria*, *Leucosporidium*, *Stellatospora*, *Serendipita*, *Haliangium*, *Luteitalea*, *Shermanella* and *Tahibacter*. For leeks and clover, AMF could be combined with *Acremonium*, *Buergenerula*, *Serendipita*, *Gaiella*, *Pseudomonas*, *Streptomyces* and *Terrimonas*. The inoculum for clover and other legumes could also include *Agromyces* and *Rhizobium*.

However, translating these network-derived candidates into robust, multi-partner inocula is constrained by both methodological and taxonomic limitations. The main challenge in designing multi-partner microbial inocula is the limited cultivability of soil microorganisms under controlled laboratory conditions. In addition, accurately identifying soil microorganisms at the species level remains difficult, which further complicates the selection and assembly of effective microbial consortia. For example, the majority of AM fungal species recorded in our dataset could only be reliably identified at the genus level. A BLAST search of AMF sequences against the SILVA, Genbank (Benson et al. 2013) and the AMF-specific MaarjAM database (Öpik et al. 2010) rarely yielded unambiguous species names. Because AMF rDNA copies can be highly variable at the intragenomic level, it can blur the boundary between intra- and interspecific variation, and no uniform distance thresholds can be considered to infer conspecificity with database sequences (Stefani et al. 2025). Similarly, fungal OTUs and bacterial ASVs were mostly resolved to the genus level, with few species identified. Other limitations also arise from the implementation and study of co-occurrence network analyses. The technical limitations of sequencing devices and data processing after sequencing can influence co-occurrence network structure and interpretation (Goberna and Verdú 2022; Oña et al. 2025). Moreover, the ecological nature (mutualism, commensalism, antagonism, parasitism, etc.) of positive or negative co-occurrences is not directly inferred for several reasons. Firstly, determining the type of ecological interactions requires experimental studies or background information (Goberna and Verdú 2022). Moreover, co-occurrence networks are based on patterns of symmetrical correlations, derived from correlation tests (e.g., Pearson correlation) doing pairwise comparisons, whereas most ecological interactions are inherently asymmetrical (Carr et al. 2019; Li et al. 2023). Indeed, interactions between different taxa are affected by the differences in relative abundances between rare taxa and abundant taxa. Then, improved taxonomic resolution and experimental analyses are therefore essential to validate predicted interactions and refine AMF-based inocula. Candidate consortia should be evaluated under controlled conditions for their effects on plant growth, health and stress tolerance (Lebeis 2014). However, the isolation and cultivation of the majority of soil microorganisms is recalcitrant to in vitro culture on synthetic media (Pham and Kim 2012; Basu et al. 2015). Field trials are also necessary to evaluate inoculum stability, agronomic performance and resilience in real conditions (Neuhoff et al. 2024), with adjustments made iteratively based on the plant response and microbial establishment (Khare and Arora 2015). Since AMF strains can adapt to specific soils and climates (Díaz-Rodríguez et al. 2025) and compete with native microorganisms, including local AMF (Graham 2001; Trabelsi and Mhamdi 2013; Thomsen et al. 2021), using locally derived strains, such as those identified in this study, will likely lead to more successful inoculation.

## Conclusion

The present study highlights the complex relationships between AMF, bacteria and non-*Glomeromycotina* fungi. Our results showed that microbes’ diversity and interactions between root-associated microorganisms could vary depending on the plant species and ecological niche. Co-occurrence networks are key tools for investigating positive interactions among AMF, beneficial bacteria and non-*Glomeromycotina* fungi. These interactions should provide concrete guidance for designing microbial inocula that are either generalist or host specific. This study was conducted with crops grown under semi-controlled conditions, where the influence of the environment on the plant development and their microbial communities is minimal. Future research should first reproduce the same results from biological samples coming from field crops. Future research should also experimentally validate these network-based results and address current limitations in culturing key soil microorganisms. Finally, the optimisation of microbial consortia for different agricultural conditions should be carried out so that multi-partner inocula can be used in sustainable agricultural practices.

## Supplementary Information

Below is the link to the electronic supplementary material.

Supplementary Material 1

## Data Availability

The Illumina data generated in this study were deposited in the NCBI Sequence Read Archive and are available under project number PRJNA1368353.
