# Supplementary material for: Co-occurrence networks reveal candidate AMF–microbe assemblages for generalist and crop-specific inocula
Source: Mycorrhiza. 2026 Apr 13;36(2):14. doi: 10.1007/s00572-026-01257-4 (PMC13076415; doi:10.1007/s00572-026-01257-4)
Supplement: Supplementary file 1 — Supplementary Material 1 [file 572_2026_1257_MOESM1_ESM.pdf]

Co-occurrence networks reveal candidate AMF–microbe assemblages for generalist and crop-specific inocula

Journal: *Mycorrhiza*

Mathieu Delaeter,[1] Ndour Papa Mamadou Sitor,[1] Benoit Tisserant,[1] Béatrice Randoux,[1] Lobna Abdellatif,[2] Franck Stefani,[2] Maryline Magnin-Robert,[1] Anissa Lounès-Hadj Sahraoui[1,*]

[1] Unité de Chimie Environnementale et Interactions sur le Vivant (UCEIV, UR 4492), Université du Littoral Côte d'Opale, 50 rue Ferdinand Buisson, 62228 Calais CEDEX, France.

[2] Ottawa Research and Development Centre, Agriculture and Agri-Food Canada, Ottawa, ON, Canada.

* Corresponding author. E-mail addresses: anissa.lounes@univ-littoral.fr (A. Lounes-Hadj Sahraoui).

Online Resource, Table 1

Table 1 GPS coordinates of A, B and C soils sampling locations.

Samples	GPS coordinates
**A**	50°24′42.6″ N 2°31′10.4″ E
**B**	50°24′30.993″ N 2°31′13.575″ E
**C**	50°24′39.815″ N 2°31′37.236″ E

Online Resource, Table 2

Table 2 Soil physical and chemical parameters have been measured. For each parameter, each value is the mean of two technical replicates values. No significant differences among treatments were detected (Kruskal–Wallis test, followed by Dunn's post hoc test, $p > 0.05$).

Samples	pH in water	Organic matter ($g.kg^{-1}$)	Organic carbon ($g.kg^{-1}$)	Nitrogen ($g.kg^{-1}$)	Ratio C/N	Carbonates ($g.kg^{-1}$)	Phosphorus Olsen ($g.kg^{-1}$)	Potassium ($g.kg^{-1}$)	Calcium ($g.kg^{-1}$)	Magnesium ($g.kg^{-1}$)	Cation Exchange Capacity ($meq.kg^{-1}$)
**A**	8.08	27.09	15.76	1.52	9.50	24.50	0.08	0.21	6.75	0.09	98.77
**B**	7.93	31.45	18.28	1.69	10.00	12.50	0.11	0.26	4.37	0.10	109.46
**C**	8.10	25.53	14.84	1.47	9.50	17.00	0.08	0.31	4.71	0.09	96.82
**Mean**	8.04 (±0.09)	28.02 (±3.07)	16.29 (±1.78)	1.56 (±0.11)	9.67 (±0.29)	18.00 (±6.06)	0.09 (±0.02)	0.26 (±0.05)	5.28 (±1.29)	0.09 (±0.005)	101.68 (±6.81)

Online Resource, Figure 1

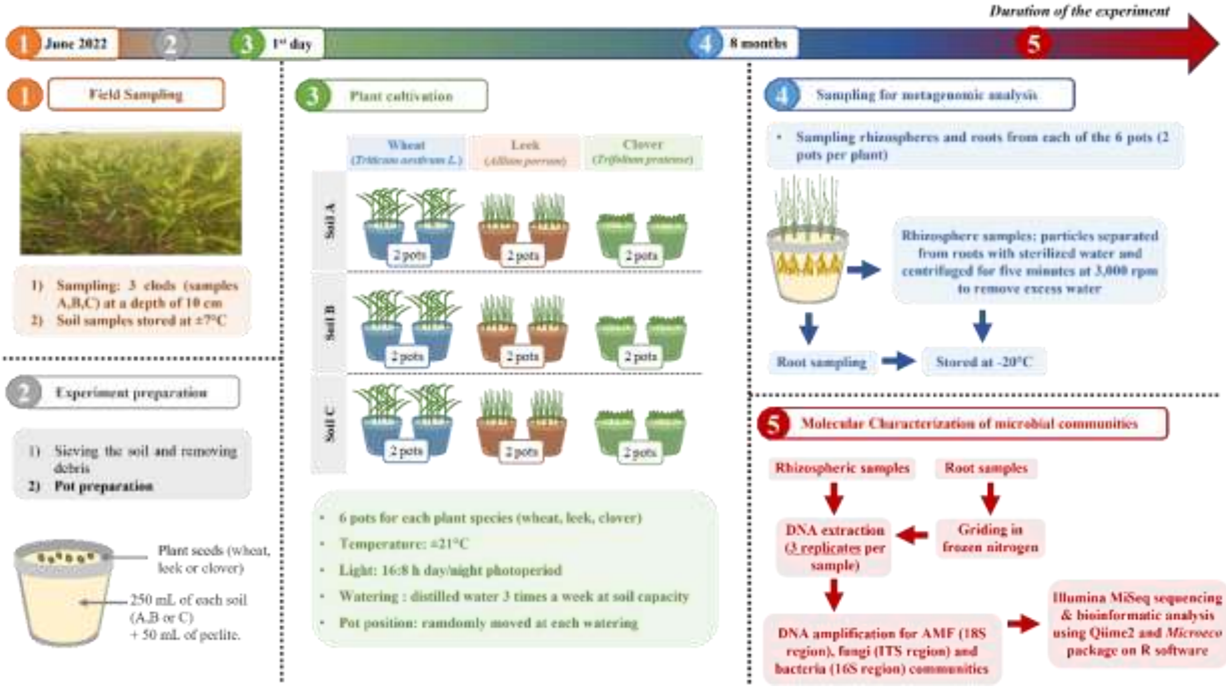

Figure 1 Experimental design and sampling.

Online Resource, Table 3

Table 3 Degenerate fusion primers used for the nested PCR. The Illumina adapters and primers are shown in black and red, respectively. These primers were used as a pool to amplify the V3-V4 fragment of the 18S rRNA genes during the second-round PCR. A 1–3 bp “heterogeneity spacer” (N, NN and NNN) was introduced between the 3′ end of the adapter and the 5′ end of the primer pair nu-SSU-0450-5′/nu-SSU-0899-3′ to mitigate the low sequence diversity issue on the MiSeq platform (Fadrosh et al. 2014).

Primer name	Sequence (5′ – 3′)
Illu_Rd1_0N_nu-SSU-0450-5’	TCGTCGGCAGCGTCAGATGTGTATAAGAGACAGCGCAAATTACCCAATCCC
Illu_Rd1_1N_nu-SSU-0450-5’	TCGTCGGCAGCGTCAGATGTGTATAAGAGACAGNCGCAAATTACCCAATCCC
Illu_Rd1_2N_nu-SSU-0450-5’	TCGTCGGCAGCGTCAGATGTGTATAAGAGACAGNNCGCAAATTACCCAATCCC
Illu_Rd1_3N_nu-SSU-0450-5’	TCGTCGGCAGCGTCAGATGTGTATAAGAGACAGNNNCGCAAATTACCCAATCCC
Illu_Rd1_0N_nu-SSU-0899-3’	GTCTCGTGGGCTCGGAGATGTGTATAAGAGACAGATAAATCCAAGAATTTCACCTC
Illu_Rd1_1N_nu-SSU-0899-3’	GTCTCGTGGGCTCGGAGATGTGTATAAGAGACAGNATAAATCCAAGAATTTCACCTC
Illu_Rd1_2N_nu-SSU-0899-3’	GTCTCGTGGGCTCGGAGATGTGTATAAGAGACAGNNATAAATCCAAGAATTTCACCTC
Illu_Rd1_3N_nu-SSU-0899-3’	GTCTCGTGGGCTCGGAGATGTGTATAAGAGACAGNNNATAAATCCAAGAATTTCACCTC

Online Resource, Figure 2

Figure 2 Alpha diversity (Hill numbers) of AMF, fungi, and bacteria in rhizosphere and roots of wheat (W), leek (L), and clover (C). Boxplots show the distribution of Species richness (q=0) (top panels), exponential Shannon entropy (q=1) (middle panels) and inverse Simpson index (q=2) (bottom panels) for each plant–niche combination. In each boxplot, the central line represents the median, the box spans the interquartile range (IQR, 25th–75th percentile), and the whiskers extend to 1.5 × IQR; points beyond the whiskers indicate outliers. Y-axes are scaled independently in each panel to enhance the visibility of differences among treatments. Asterisks above the boxplots indicate significant differences between plant species, as determined by Kruskal-Wallis test followed by Dunn test (α = 0.05). Significance levels are indicated as follows: * for p ≤ 0.05, ** for p ≤ 0.01, *** for p ≤ 0.001 and **** for p ≤ 0.0001.

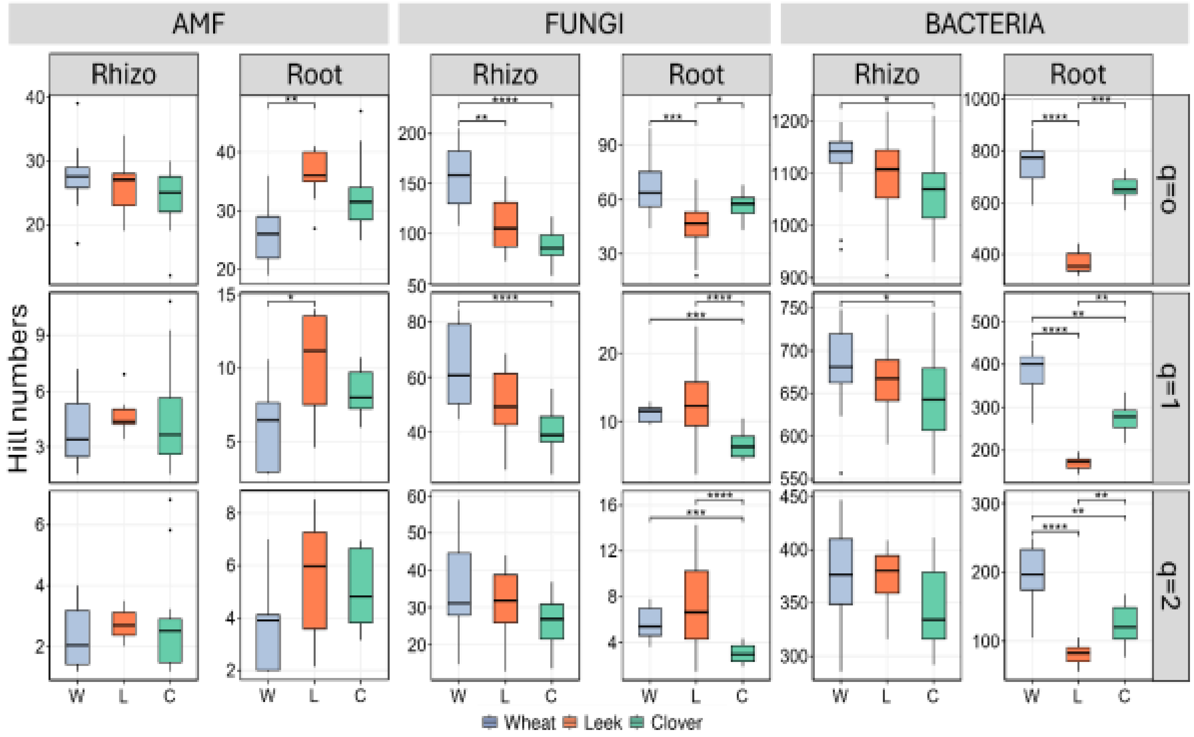

Online Resource, MiSeq dataset features

Three datasets were obtained after Illumina MiSeqTM sequencing. From nu-SSU raw dataset (AMF), ITS raw dataset (fungi) and 16S raw dataset (bacteria), a total of 10,073,763 sequences (449 to 456 bp in length), 4,198,605 sequences (281 to 483 bp in length) and 4,088,465 sequences (371 to 375 bp in length) were obtained, respectively. For AMF, bacterial and fungal sequencing datasets, the denoising procedure reduced the number of sequences to 3,935,998-3,302,327-2,609,185 sequences, respectively, resulting in a total of 1,640 ASVs, 3,857 OTUs and 24,478 ASVs. This number of representative sequences was further reduced to 1,098 ASVs, 2,760 OTUs and 7,019 ASVs after the elimination of rare ASVs. De novo clustering reduced the number of AMF and fungal representative sequences to 131 ASVs and 992 OTUs, respectively. After final rarefaction, the sequence libraries were composed of 1,460,000 sequences across 73 samples and 131 ASVs for AMF community, 1,030,000 sequences across 103 samples and 992 OTUs for fungal community and 2,645,964 sequences across 98 samples and 8,628 ASVs for bacterial community.

Online Resource, Table 5

Table 5 Table referencing positive interactions found between different genera of AMF and non-*Glomeromycotina* fungi.

Fungal phyla	Fungal orders	Fungal genera	Rhizosphere														Roots												
			Wheat				Leek						Clover				Wheat					Lee	Clover						
			Diversispora	Other Diversisporales genera	*Funneliformis*	*Scutellospora*	*Archaeospora*	*Acaulospora*	*Diversispora*	*Glomus*	*Scutellospora*	*Septoglomus*	*Archaeospora*	*Diversispora*	*Glomus*	*Rhizophagus*	*Diversispora*	*Funneliformis*	*Glomus*	*Septoglomus*	*Unidentified*	*Glomus*	*Diversispora*	Other Diversisporales genera	*Entrophospora*	*Funneliformis*	*Glomus*	*Rhizophagus*	*Septoglomus*
Ascomycota	Ascomycota_ord_Incertae_sedis	*Ascomycota_gen_Incertae_sedis*	1										1											2	1				
	Cladosporiales	*Cladosporium*																1								1			
	Eurotiales	*Penicillium*											1			3													
	Glomerellales	*Sodiomyces*					1																						
	Helotiales	*Cadophora*																									1		
		Hymenoscyphus																							1				
		Oidiodendron													2														
	Hypocreales	*Acremonium*						1					1																
		Eucasphaeria							1	1	4																		
		Fusarium							1	1	4												1	1	3		4	1	
		Fusicolla															1												
		Lasionectria				1																							
		Sarocladium																				3							
		Stachybotrys										1																	
		Trichoderma					1																						
		Unidentified																				1		1					
	Magnaporthales	*Buergenerula*						1					1																
	Microascales	*Cephalotrichum*	1			2																							
		Enterocarpus											1		2	3													
	Microthyriales	*Antidactylaria*													1														
	Mycosphaerellales	*Unidentified*																		1									
	Orbiliales	*Arthrobotrys*																									1		
	Pezizales	*Ascobolus*																									2	1	
		Cheilymenia	1																										
		Scutellinia					1																						
	Pleosporales	*Clohesyomyces*																			1								
		Paraphaeosphaeria						1																					
		Pleosporales_gen_Incertae_sedis					1																						
		Unidentified																					2	1	1				
	Sordariales	*Lasiosphaeriaceae_gen_Incertae_sedis*						1																					
		Neoschizothecium						1											1										
		Ramophialophora																				1							
		Schizothecium												1															
		Stellatospora			1																								
		Sordariales_gen_Incertae_sedis																									1		
		Unidentified					1																						
	Xylariales	*Fusidium*	1										1															2	
		Microdochium											1			3													

	Unidentified	***Unidentified***		1					1		4																		
**Basidiobolomycota**	**Basidiobolomycota_ord_Incertae_sedis**	***Basidiobolomycota_gen_Incertae_sedis***							1	1	4				1														
**Basidiomycota**	**Agaricales**	***Coprinopsis***											2														1		
		Entoloma	1																										
		Psathyrella							1	1	4																		
	**Auriculariales**	***Unidentified***	1																										
	**Cantharellales**	***Ceratobasidium***																				3							
	**Corticiales**	***Corticiaceae_gen_Incertae_sedis***							1	1	4																		
	**Helicobasidiales**	***Helicobasidiaceae_gen_Incertae_sedis***																					1						1
	**Leucosporidiales**	***Leucosporidium***	1																										
	**Platygloeales**	***Eocronartium***											1	1															
	**Sebacinales**	***Serendipita***							1											1		3	1		1		4	1	
	**Unidentified**	***Unidentified***																1											
**Chytridiomycota**	**Chytridiomycota_ord_Incertae_sedis**	***Chytridiomycota_gen_Incertae_sedis***													2														
	**Cladochytriales**	***Nowakowskiella***																									2		
	**Lobulomycetales**	***Lobulomycetales_gen_Incertae_sedis***										1																	
	**Lobulomycetes_ord_Incertae_sedis**	***Lobulomycetes_gen_Incertae_sedis***										1																	
	**Rhizophydiales**	***Protrudomyces***						1																					
		Rhizophydiales_gen_Incertae_sedis						1																					
**Kickxellomycota**	**Kickxellales**	***Coemansia***													2														
**Mortierellomycota**	**Mortierellales**	***Mortierella***																					1		1				

Online Resource, Table 6

Table 6 Table referencing microbial functions from non-*Glomeromycotina* fungal genera interacting with AMF.

Genera	Soil saprotroph	Wood saprotroph	Litter saprotroph	Endophyte	Plant pathogen	Parasite	Identified species
Acremonium	x						*Acremonium rutilum*
Antidactylaria		x					*Antidactylaria ampulliformis*
Arthrobotrys	x			x			*Arthrobotrys reticulatus*
Ascobolus	x						
Buergenerula				x			
Cadophora				x			
Cephalotrichum	x	x					*Cephalotrichum stemonitis*
Ceratobasidium				x	x		
Cheilymenia			x				*Cheilymenia stercorea*
Cladosporium	x	x	x	x			*Cladosporium herbarum*
Clohesyomyces			x	x			
Coemansia			x				*Coemansia helicoidea*
Coprinopsis	x			x			
Enterocarpus	x						*Enterocarpus grenotii*
Entoloma	x						
Eocronartium						x	*Entoloma punjabense*
Eucasphaeria		x	x				*Eucasphaeria capensis*
Fusarium					x		*Fusarium graminearum; Fusarium solani; Fusarium venenatum*
Fusicolla		x		x			*Fusicolla septimanifiniscientiae*
Fusidium	x			x			
Hymenoscyphus		x		x			*Hymenoscyphus menthae*
Lasionectria	x						*Lasionectria hilhorstii*
Leucosporidium	x						
Microdochium					x		
Mortierella	x			x			

Neoschizothecium	x						*Neoschizothecium carpinicola; Neoschizothecium curvisporum*
Nowakowskiella	x						*Nowakowskiella elegans*
Oidiodendron				x			
Paraphaeosphaeria	x						
Penicillium	x						
Protrudomyces	x						*Protrudomyces lateralis*
Psathyrella	x						*Psathyrella sacchariolens*
Ramophialophora	x						*Ramophialophora humicola*
Sarocladium				x	x		*Sarocladium strictum*
Schizothecium			x	x			
Scutellinia		x					
Serendipita				x			
Sodiomyces	x						
Stachybotrys		x					*Stachybotrys limonisporus*
Stellatospora	x						*Stellatospora erricola*
Trichoderma	x						

Online Resource, Table 7

Table 7 Table referencing positive interactions found between different genera of AMF and bacteria.

Bacterial phyla	Bacterial orders	Bacterial genera	Rhizosphere					Roots															
			Leek			Clover		Wheat						Leek				Clover					
			Diversispora	Other Diversisporales genera	*Entrophospora*	*Acaulospora*	*Diversispora*	*Diversispora*	Other Diversisporales genera	*Funneliformis*	*Glomus*	*Rhizophagus*	*Unidentified*	Other Diversisporales genera	*Entrophospora*	*Glomus*	*Rhizophagus*	*Diversispora*	Other Diversisporales genera	*Entrophospora*	*Glomus*	*Rhizophagus*	*Septoglomus*
Acidobacteriota	Subgroup	*Subgroup*																1		1			
	Thermoanaerobaculales	*Subgroup*									3					3	1			1			
	Vicinamibacterales	*Luteitalea*						1															
		Unidentified						1															
Actinobacteriota	Gaiellales	*Gaiella*														2			1				
	Kineosporiales	*Kineosporia*																				1	
	Micrococcales	*Agromyces*																	2	1			
		Unidentified												1							1		
	Micromonosporales	*Actinoplanes*																1		1	5	1	
		Dactylosporangium																				1	
		Unidentified																1			3	2	
	Microtrichales	*Uncultured*																			5	1	
	Propionibacteriales	*Nocardioides*															1						
	Pseudonocardiales	*Pseudonocardia*								2						1							
	Streptomycetales	*Streptomyces*													2		2			1			
Bacteroidota	Bacteroidales	*WCHB*		1																			
	Chitinophagales	*Terrimonas*														1					1		
		Uncultured																		1	6	2	
		Unidentified																			5		
	Cytophagales	*Chryseolinea*											1									1	
		Dyadobacter																	2			1	
		Unidentified													1								
	Flavobacteriales	*Chryseobacterium*																	1				
		Flavobacterium																		1	1		
	Kryptoniales	*BSV*		1																			
	Sphingobacteriales	*Mucilaginibacter*																	1	2			
Chloroflexi	Anaerolineales	*Unidentified*		1																			
		UTCFX																				2	
	Ardenticatenales	*Uncultured*											1										
	Chloroflexales	*Candidatus Chloroploca*														2							
		Uncultured								1													
	Kallotenuales	*AKIW*				1															12		
	SBR	*Ab*														1					2		
Crenarchaeota	Nitrososphaerales	*Nitrososphaeraceae*																		1			
		Candidatus Nitrocosmicus					1																
Desulfobacterota	Desulfovibrionales	*Desulfovibrio*								1													
Fibrobacterota	Fibrobacterales	*Possible genus*											1										
Firmicutes	Bacillales	*Unidentified*	1																				

	**Clostridia**	***Unidentified***		1												1							
**Gemmatimonadota**	**Longimicrobiales**	***Longimicrobium***																1					
**Myxococcota**	**Haliangiales**	***Haliangium***								1	1												
	**Myxococcales**	***Unidentified***														1							
**Planctomycetota**	**Pirellulales**	***Unidentified***										1											
**Proteobacteria**	**Azospirillales**	***Skermanella***								1													
	**Burkholderiales**	***Duganella***																	2	1			1
		Ellin																1	1	1	1	2	
		Massilia																1	3	1			1
		uncultured														1							
		Unidentified	1		1					2	1	1					2		8	2	9		
	**Caulobacterales**	***Caulobacter***																	1	1	5	1	
	**Enterobacterales**	***Unidentified***																			1	1	
	**Gammaproteobacteria Incertae Sedis**	***Acidibacter***							1														
	**Pseudomonadales**	***Pseudomonas***													1			1	1		5		
		Uncultured													2								
	**Rhizobiales**	***Allorhizobium, Neorhizobium, Pararhizobium, Rhizobium***																			5		
		Uncultured														1							
		Unidentified																		1		1	
	**Sphingomonadales**	***Novosphingobium***															1				1		
		Sphingomonas																				1	
	**Xanthomonadales**	***Dokdonella***																	2				
		Lysobacter																	1				
		Pseudoxanthomonas																				1	
		Stenotrophomonas																			1	1	
		Tahibacter									1									1			
		Unidentified														1					1		
**Verrucomicrobiota**	**Opitutales**	***Opitutus***																			5	1	

Online Resource, Table 8

Table 8 Table referencing microbial functions from bacterial genera interacting with AMF.

Genera	Saprophyte	NFB	Nitrification	Denitrification	PSB	PGPR	Plant pathogen	MHB	AMF symbiont	Identified species
Ab										
Acidibacter	x									
Actinoplanes	x								x	
Agromyces	x	x							x	
AKIW										
Allorhizobium, Neorhizobium, Pararhizobium, Rhizobium		x				x		x	x	
BSV										
Candidatus Chloroploca										
Candidatus Nitrocosmicus										
Caulobacter						x			x	
Chryseobacterium						x	x		x	
Chryseolinea									x	
Dactylosporangium	x								x	
Desulfovibrio									x	*Desulfovibrio putealis*
Dokdonella				x					x	
Duganella					x	x			x	
Dyadobacter						x			x	*Dyadobacter endophyticus*
Ellin										
Flavobacterium					x	x			x	
Gaiella	x								x	
Haliangium						x			x	
Kineosporia									x	
Longimicrobium	x								x	
Luteitalea	x									
Lysobacter		x				x			x	
Massilia						x		x	x	
Mucilaginibacter	x					x			x	
Nitrososphaeraceae genus			x							
Nocardioides	x								x	
Novosphingobium	x								x	
Opitutus	x								x	
Pseudomonas				x	x	x		x		*Pseudomonas mandelii*
Pseudonocardia						x				
Pseudoxanthomonas						x			x	
Skermanella									x	*Skermanella aerolata*
Sphingomonas					x	x			x	
Stenotrophomonas					x	x			x	
Streptomyces	x	x						x	x	
Tahibacter									x	
Terrimonas									x	*Terrimonas crocea*
UTCFX										
WCHB										
